# Effects of Long-Term Mindfulness Meditation on Brain's White Matter Microstructure and its Aging

**DOI:** 10.3389/fnagi.2015.00254

**Published:** 2016-01-14

**Authors:** Davide Laneri, Verena Schuster, Bruno Dietsche, Andreas Jansen, Ulrich Ott, Jens Sommer

**Affiliations:** ^1^Department of Psychiatry and Psychotherapy, University of MarburgMarburg, Germany; ^2^Department of Psychology, Bender Institute of Neuroimaging, Justus-Liebig-University GiessenGiessen, Germany

**Keywords:** DTI, meditation, thalamus, insula, amygdala, hippocampus, ACC, aging

## Abstract

Although research on the effects of mindfulness meditation (MM) is increasing, still very little has been done to address its influence on the white matter (WM) of the brain. We hypothesized that the practice of MM might affect the WM microstructure adjacent to five brain regions of interest associated with mindfulness. Diffusion tensor imaging was employed on samples of meditators and non-meditators (*n* = 64) in order to investigate the effects of MM on group difference and aging. Tract-Based Spatial Statistics was used to estimate the fractional anisotrophy of the WM connected to the thalamus, insula, amygdala, hippocampus, and anterior cingulate cortex. The subsequent generalized linear model analysis revealed group differences and a group-by-age interaction in all five selected regions. These data provide preliminary indications that the practice of MM might result in WM connectivity change and might provide evidence on its ability to help diminish age-related WM degeneration in key regions which participate in processes of mindfulness.

## Introduction

Although mindfulness meditation (MM) was born as one of the main aspects of the Buddhist tradition, in recent years it has been generally taught in Western countries independently of any religion. A modern definition of mindfulness recently formulated by Kabat-zinn (2003), states that the practice of mindfulness involves the purposeful focusing of one's attention in the present moment with a nonjudgmental attitude. In the last few years there has been an increasing interest in meditation and mindfulness in various fields. Latest clinical studies, for example, have largely focused on MM for the treatment of patients with depression, anxiety, stress, or pain, (Chiesa and Serretti, 2009, 2010, 2011; Hofmann et al., 2010; Keng et al., 2011; Cramer et al., 2012; Eberth and Sedlmeier, 2012; Mitchell et al., 2013; Goyal et al., 2014). Moreover, it has been shown that MM may contribute to improve cognitive performances such as attention, memory, and concentration (Jha et al., 2007; Tang et al., 2007; Lutz et al., 2009; MacLean et al., 2010; Chiesa et al., 2011; Greenberg et al., 2012; Mrazek et al., 2013). Furthermore, there is a growing body of evidence demonstrating how mindfulness and meditation affect physiological and biological body and brain properties such as telomere elongation, hemodynamics, and cerebral blood flow (Solberg et al., 2004; Khalsa et al., 2009; Newberg et al., 2010; Hoge et al., 2013; Jacobs et al., 2013). When considering the results of these studies, it comes almost naturally to ask whether MM also has an impact on the structure of the brain. The utilization of magnetic resonance imaging (MRI) techniques has provided some evidence in this regard. In a recent meta-analysis Fox et al. (2014) carried out a systematic review and meta-analysis of 21 morphometric neuroimaging studies in meditation practitioners. They found eight brain regions consistently altered in meditators. Seventeen of the 21 articles reviewed, investigated the effects of MM on gray matter (GM) macrostructure. Most of them utilized voxel based morphometry (VBM) and demonstrated that meditators, compared with non-meditators, show increased regional GM volumes and thicker cortical areas. For instance, Lazar et al. (2005) observed that meditators had a thicker prefrontal cortex as well as a thicker anterior insula. Moreover, Luders et al. (2009) found larger GM density in the orbitofrontal cortex and in the right hippocampus. Hölzel et al. (2008) detected higher GM matter concentration for meditators in the right anterior insula, left inferior temporal gyrus and right hippocampus. In addition, Hölzel et al. (2011) also found increased GM concentration within the left hippocampus, the posterior cingulate cortex, the temporo-parietal junction and the cerebellum after 8 weeks of mindfulness meditation practice.

However, these studies only focused on GM differences, lacking insight into the impact of MM on white matter (WM) microstructure. Only a very small number of diffusion tensor imaging (DTI) studies exist which have demonstrated how the practice of meditation may be responsible for an increase of WM connectivity and integrity parameters, such as fractional anisotropy (FA) and mean diffusivity (MD), in several white matter tracts (Tang et al., 2010, 2012a; Luders et al., 2011, 2012b; Fayed et al., 2013; Kang et al., 2013). Although reporting promising results, these studies suffer from several shortcomings. They have either only carried out a whole brain analysis (Tang et al., 2010, 2012a; Kang et al., 2013), only investigated structural changes of main tracts of the WM (Luders et al., 2011, 2012b) or their focus was not primarily on DTI (Fayed et al., 2013; for an overview see Fox et al., 2014). However, none of these studies have examined whether the WM directly connected to specific brain areas which are related to mindfulness show differences in fiber structure. In the present study, the regions of interest (ROI) were selected according to previous results from structural and functional studies in which FA values, brain regions volume or activation was found to be altered for meditation practitioners (Lazar et al., 2005; Hölzel et al., 2008, 2010, 2011; Luders et al., 2009, 2012a, 2013a,b; Chiesa and Serretti, 2010; Grant et al., 2010; Newberg et al., 2010; Murakami et al., 2012; Tang et al., 2012b; Fayed et al., 2013; Lutz et al., 2013a; Mascaro et al., 2013; Taren et al., 2013). Specifically, the anterior cingulate cortex (ACC), the insula, the amygdala, the thalamus, and the hippocampus were chosen based on previous results which suggest their significant influence in emotion regulation, attention, self awareness, pain regulation, and perspective taking. In Fox et al. (2014) for example, all the above mentioned ROIs or the WM connecting them are included in the results of several of the selected morphometric studies. They often also appear to be part of functional meditation studies outcomes displaying different activation patterns in meditators (Farb et al., 2007, 2012; Goldin and Gross, 2010; Taylor et al., 2011; Zeidan et al., 2011, 2014; Desbordes et al., 2012; Gard et al., 2012; Pickut et al., 2013; Lutz et al., 2014).

In addition, not much has been done in order to ascertain the impact of mindfulness on the decline of structural WM parameters due to aging. To our knowledge, only three studies and one recent meta-analysis (which has reviewed their findings) have addressed this topic (Lazar et al., 2005; Pagnoni and Cekic, 2007; Luders et al., 2011; Luders, 2014). Considering that brain volume, density and integrity generally decrease and brain atrophy generally increases with age (Michielse et al., 2010; Lebel et al., 2012; Sala et al., 2012; Voineskos et al., 2012), it seems appropriate to further investigate whether MM contributes to a slowing down or whether it even protects the brain, or some of its regions, from the aging process.

The purpose of this cross-sectional study was to compare MM practitioners with controls in order to investigate differences in WM integrity and age-related decline.

First we employed a DTI approach on specific ROIs to analyze the WM microstructure directly connected to MM related areas. Secondly, we investigated whether meditators differed from non-meditators with respect to the effect of aging on the WM connected to the selected ROIs. Based on the aforementioned studies we expected MM to influence the age-related decline of brain WM parameters and plasticity by slowing down the natural decrease of FA measures in the WM adjacent to the thalamus, insula, amygdala, hippocampus, and ACC. We also expected to observe structural differences between groups in the WM around the selected ROIs.

## Methods

### Participants

Thirty-three healthy subjects who regularly practice meditation (“meditators”), recruited from Buddhist and Zen centers all over Germany, were included in the final analysis of this study. Meditators reported to live an ordinary life involving family, career and other common activities. Meditation experience among meditators ranged between 5 and 38 years. All meditators reported a regular practice of MM styles such as Vipassana, Shamatha, and Zazen. For the control group, thirty-one healthy subjects with no meditation experience (“non-meditators”) were included. Both groups did not differ in terms of sex, age, handedness, and IQ (see Table 1). Exclusion criteria were identified as follows: diagnosed neurological or psychiatric disorders (present and past); current alcohol or drug abuse, use of psychiatric medications (present and past); anatomical brain abnormalities (e.g., lesions, strokes etc.). This study was carried out in accordance with the recommendations of the Declaration of Helsinki, and the experimental protocol was approved by the ethical committee of the University of Marburg (Faculty of Medicine). All subjects gave written informed consent.

**Table 1:** **Socio-demographic characteristics of both meditators (MED) and non-meditators (CON)**.

	**MED (*n* = 33) M (SD)**	**CON (*n* = 31) M (SD)**	**Statistic**	***P***
Age	51.42 (7.64)	50.09 (5.63)	*t* = 0.79	0.44
Gender (m/f)	22/11	19/12	*x*^2^ = 0.20	0.65
Estimated IQ^a^^,^ ^b^	33.43 (1.86)	33.53 (2.02)	*t* = 0.12	0.84
Handedness (r/l)	30/3	30/1	*x*^2^ = 0.16	0.69
Meditation Exp. (yrs)	17.27 (9.79)	–	–	–

*M, mean; SD, standard deviation*.

a *Assessed with a multiple vocabulary test (Lehrl et al., 1995)*.

b *Available for n = 62*.

### Data acquisition

DTI measurements were performed on a 3-T MRI (Tim Trio; Siemens, Erlangen, Germany) for all participants. All diffusion-weighted images were acquired using a singleshot echo-planar imaging sequence (repetition time, 7300 ms; echo time, 90 ms; field of view, 256 mm; matrix, 128 × 128; slice thickness, 2.5 mm; numbers of excitations, 1; b = 1000 s/mm^2^; 30 noncollinear diffusion-encoding gradients; voxel size, 2 × 2 × 2.5 mm^3^). Two consequent sets of 30 gradients diffusion-weighted images were acquired for each participant. Acquisition time for each participant was 17 min including anatomical imaging (4 min) and diffusion weighted imaging (2 × 5 min).

Data for all participants were visually inspected for artifacts in order to identify and discard images containing subject motion. For one meditator and for one control one set of DTI data had to be discarded and for three meditators one diffusion weighted image (DWI) had to be discarded due to motion artifacts. For these participants the estimation of the diffusion tensor was based on 30 or 59, respectively, instead of 60 DWIs.

Two meditators, out of 35 initially recruited, and 1 non-meditator, out of 32 initially recruited, had to be excluded from analysis due to brain abnormalities (lesions and dissection).

The selection of candidate ROIs was based on previous findings regarding brain regions relevant to the scientific bases of mindfulness: for the thalamus (Luders et al., 2009; Newberg et al., 2010; Fayed et al., 2013; Pickut et al., 2013); for the insula, (Lazar et al., 2005; Hölzel et al., 2008, 2011; Luders et al., 2012a; Lutz et al., 2013a; Mascaro et al., 2013); for the amygdala (Hölzel et al., 2010; Desbordes et al., 2012; Murakami et al., 2012; Taren et al., 2013); for the hippocampus (Hölzel et al., 2008, 2011; Luders et al., 2009, 2013a,b); and for the ACC (Chiesa and Serretti, 2010; Grant et al., 2010; Tang et al., 2012b).

The five ROIs (all including left and right hemispheres) were extracted from the Harvard-Oxford cortical and subcortical structural atlases, which are distributed with the FSL software (http://fsl.fmrib.ox.ac.uk/fsl/fslwiki/Atlases) and are composed of well-defined brain anatomical regions. The DTI analysis was then performed on white matter areas adjacent to these regions. The investigation was not performed on separate regions, but instead the 5 ROIs were combined together to form a single mask composed of 8323 voxels used for the statistical analysis.

### Data processing

Analysis of DTI data was performed using the FMRIB Software Library (FSL) v5.02 (Smith et al., 2004) and its FMRIB's Diffusion Toolbox v3.0. Pre-processing of diffusion weighted data was performed as follows: (i) motion and eddy current corrections, (ii) removal of non-brain tissue from an image of the whole head, and (iii) fitting of diffusion tensor model at each voxel. Voxel-wise statistical analysis was performed using tract-based spatial statistics (TBSS; Smith et al., 2006) according to the following steps: (i) apply nonlinear registration of all FA images to 1 × 1 × 1 mm^3^ MNI152 standard space, (ii) creation of the mean FA image of all participants and skeletonize it, (iii) projection of all subjects' FA data onto the mean FA skeleton, setting a nonmaximum-suppression FA threshold to 0.3. The statistical analysis of the two groups was done with Randomize, also part of FSL, which enables modeling and inference using standard general linear model (GLM) design (http://fsl.fmrib.ox.ac.uk/fsl/fslwiki/Randomise).

The GLM model, used for the statistical analysis, included six contrasts (details in Supplementary Material).

For group difference, we controlled the analysis for handedness and sex, and included them in the model as variables of no interest.

A GLM analysis was carried out to study group-by-age interactions, i.e., to explore whether the slopes between FA values in the selected ROIs and age were stronger for the meditators than for controls and vice versa.

The analysis was performed using 10,000 random permutations and threshold-free cluster enhancement (TFCE; Smith and Nichols, 2009).

## Results

Results for the analysis of the two main effects (group difference and age) were corrected for multiple comparisons [familywise error rate (FWE)-corrected].

No regions/voxels from the analysis of group-by-age interaction survived FWE-corrections. Given that a regular decline in WM with age is considered to be a normal process in healthy subjects (meditators and controls both consisted of healthy individuals), interaction effects are expected to be much subtler than groups' main effects differences. Group-by-age interaction analysis was therefore repeated excluding the rather conservative FWE correction for multiple comparisons.

For the purpose of this study we only considered clusters with a minimum size of eight voxels.

Results for group difference are reported only if their clusters do not overlap with those of group-by-age interaction (e.g., with same coordinates). Overlapping voxels would clearly be a function of age.

### Group-by-age interaction

**Figure 1:**
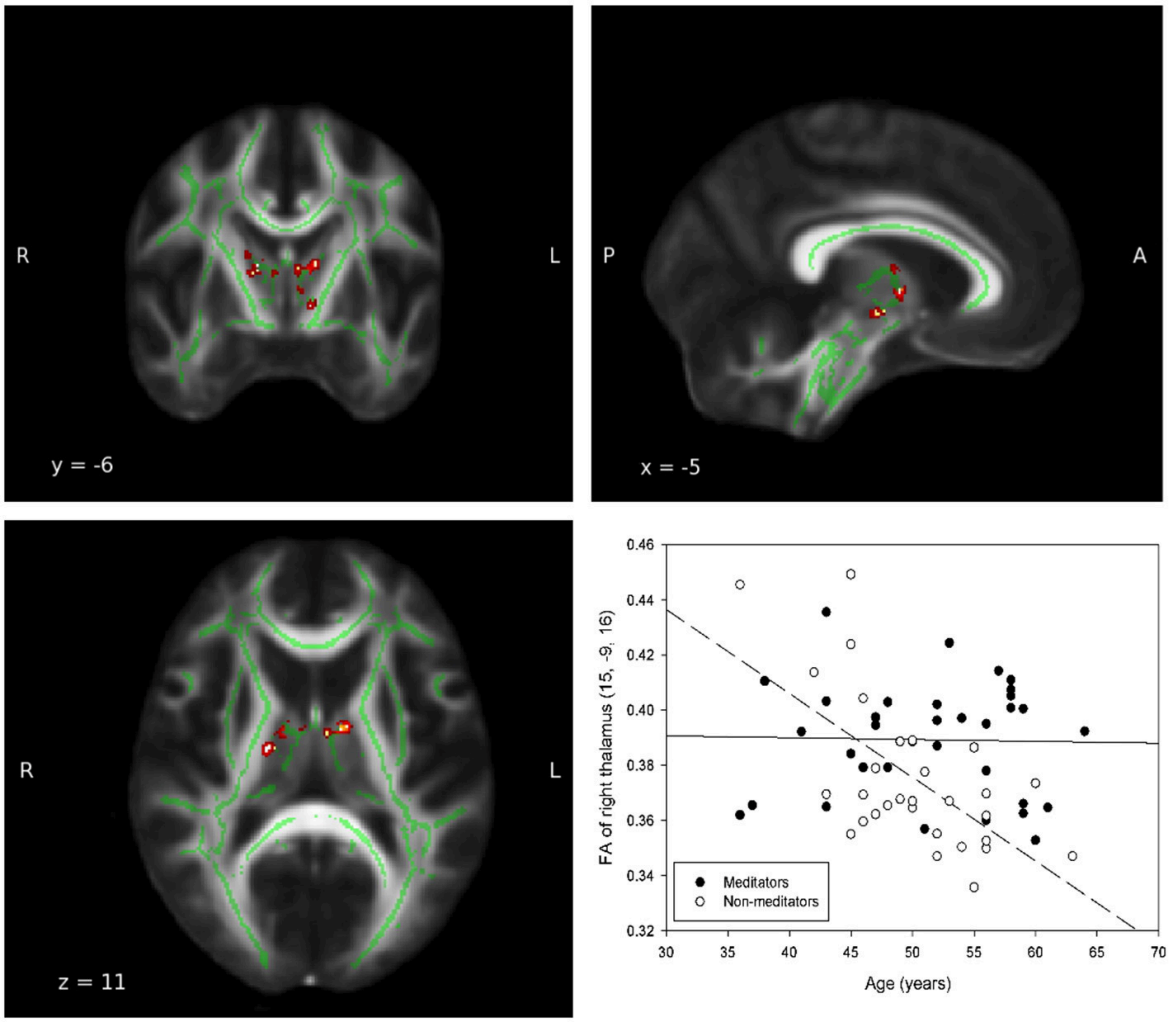
**Group-by-age interaction: Thalamus**. A significant group interaction effect between fractional anisotropy and age was detected in the white matter adjacent to the left (MNI coordinates: x = −9, y = −2, z = 9; P_uncorrected_ = 0.017) and right (MNI coordinates: x = 15, y = −9, z = 16; P_uncorrected_ = 0.002) thalamie. Coordinates (x,y, and z) are given according to the MNI coordinate system. The red and yellow areas represent the group-by-age results for the left thalamus. The green areas represent the WM skeleton of all participants. Broken regression line represents controls.

**Figure 2:**
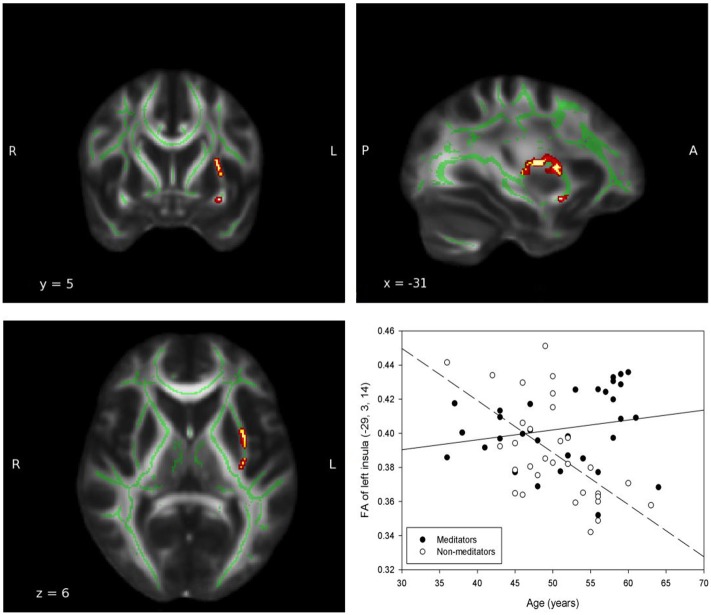
**Group-by-age interaction: Insula**. A significant group interaction effect between fractional anisotropy and age was detected in the white matter adjacent to the left insula (MNI coordinates: x = −29, y = 3, z = 14; P_uncorrected_ = 0.003). The red and yellow areas represent the group-by-age results for the left insula. The green areas represent the WM skeleton of all participants. Broken regression line represents controls.

In all ROIs, apart from right insula and left ACC, the relationship between FA, and age in non-meditators demonstrates a natural FA decline in aging adults which could not be detected in meditators (see Table 2). Two of the five ROIs that provided the strongest results are shown in Figures 1, 2.

**Table 2:** **Age interaction 1: MED > CON**.

**Anatomical region**	**Coordinates**				**Cluster**
	**x**	**y**	**z**	**No. voxels**	***P*-Value**
**THALAMUS**					
Left	−9	−2	9	51	0.017
	−16	−8	16	11	0.024
Right	15	−9	16	73	0.002
	17	−13	10	31	0.001
	5	−17	−8	12	0.027
**INSULA**					
Left	−29	3	14	202	0.003
	−26	11	−12	102	0.007
Right	–	–	–	–	–
**AMYGDALA**					
Left	−12	−10	−12	13	0.027
Right	12	−10	−12	13	0.033
**HIPPOCAMPUS**					
Left	−21	−41	−5	32	0.028
Right	9	−37	9	66	0.014
	22	−42	−1	22	0.036
**ACC**					
Left	–	–	–	–	–
Right	10	37	−17	13	0.016

*Group-by-age interaction in which meditators exhibit a weaker slope in the relationship between FA and age compared to controls (MED > CON) in left and right thalamus, left insula, left and right amygdala, left and right hippocampus and right ACC (p < 0.05, uncorrected)*.

**Figure 3:**
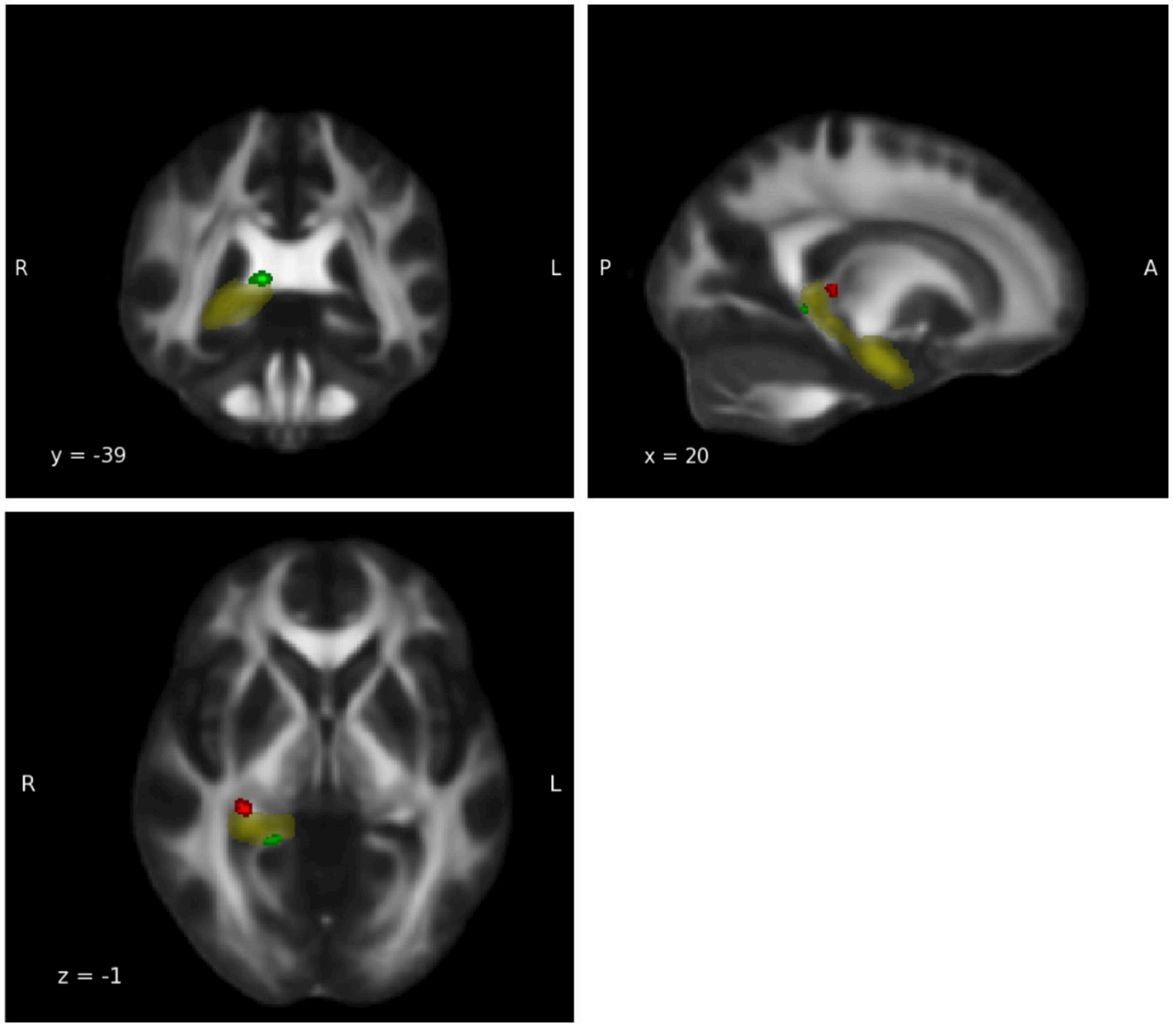
**Group-by-age interaction: Hippocampus**. Group-by-age interaction results for the hippocampus show a clear differentiation between the ventral part (red), in which meditators display a weaker negative slope, and the dorsal part (green) in which controls display a weaker negative slope (*p* < 0.05, uncorrected). Yellow: hippocampus mask from Harvard-Oxford Atlas.

For the group-by-age interaction, an opposite trend was observed in the right thalamus, the right amygdala and the right hippocampus. In the WM of these regions the expected age-related decline in FA during adulthood in the relationship between FA and age was detected in the meditators but not in the controls' group (Tables in Supplementary Material). These three ROIs therefore possess clusters in which group-by-age interaction shows both weaker and stronger slopes for the two groups. For a visual illustration of ROIs featuring both trends, see Figure 3 in which the right hippocampus was chosen as an example.

### Main effect group difference

In the right and left insula, the right thalamus and right amygdala, corrected results showed significant higher FA in meditators compared with their control group (*p* < 0.05, FWE corrected). A clear trend in the same direction was displayed by the left thalamus and right hippocampus (*p* < 0.1, FWE corrected). Uncorrected results for the left amygdala, left hippocampus and right ACC also showed a tendency toward a higher FA in meditators (*p* < 0.05, uncorrected; see Table 3). No significant clusters were found in which FA values of non-meditators were higher than FA values of meditators.

**Table 3:** **FA group difference MED > CON**.

**Anatomical region**	**Coordinates**				**Cluster**
	**x**	**y**	**z**	**No. voxels**	***P*-Value**
**THALAMUS**					
Left	−6	−4	6	23	0.075^ct^
Right	6	−12	−2	92	0.009^c^
	16	−13	14	51	0.005^c^
	9	−6	−1	22	0.04^c^
**INSULA**					
Left	−25	19	−5	153	0.005^c^
Right	30	14	−4	206	0.006^c^
**AMYGDALA**					
Left	−26	−11	−10	113	0.001^uc^
Right	33	2	−11	8	0.028^c^
**HIPPOCAMPUS**					
Left	−26	−11	−10	172	0.001^uc^
	−18	−32	3	33	0.004^uc^
Right	32	−17	−9	11	0.096^ct^
**ACC**					
Left	–	–	–	–	–
Right	21	36	17	29	0.005^uc^
	20	43	4	23	0.008^uc^
	19	45	−3	9	0.018^uc^

*Group difference in FA between meditators and controls in selected ROIs. All regions except for the ACC showed statistically significant results (p < 0.05, FWE corrected) or a clear trend (p < 0.1, FWE corrected). Uncorrected trends in the left amygdala, right hippocampus and in the right ACC are also reported (p < 0.05, uncorrected). c, corrected (p < 0.05); ct, corrected trend (p < 0.1); uc, uncorrected*.

**Figure 4:**
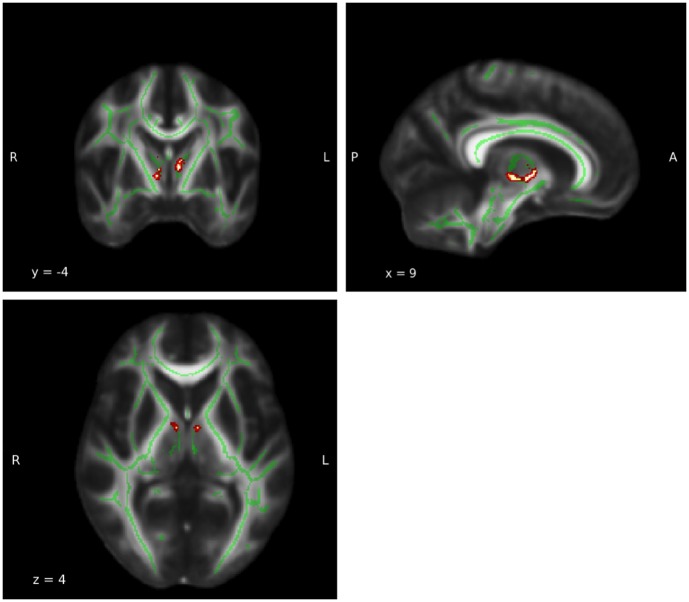
**Group difference: Thalamus**. A significant FA group difference in which the meditators displayed higher FA values was detected in the white matter adjacent to the left (MNI coordinates: x = −6, y = −4, z = 6; PFWE-corrected = 0.075) and right (MNI coordinates: x = 6, y = −12, z = −2; PFWE-corrected = 0.004) thalami. Coordinates (x, y, and z) are given according to the MNI coordinate system. The red and yellow areas represent the group difference results for the thalamus. The green areas represent the WM skeleton of all participants.

**Figure 5:**
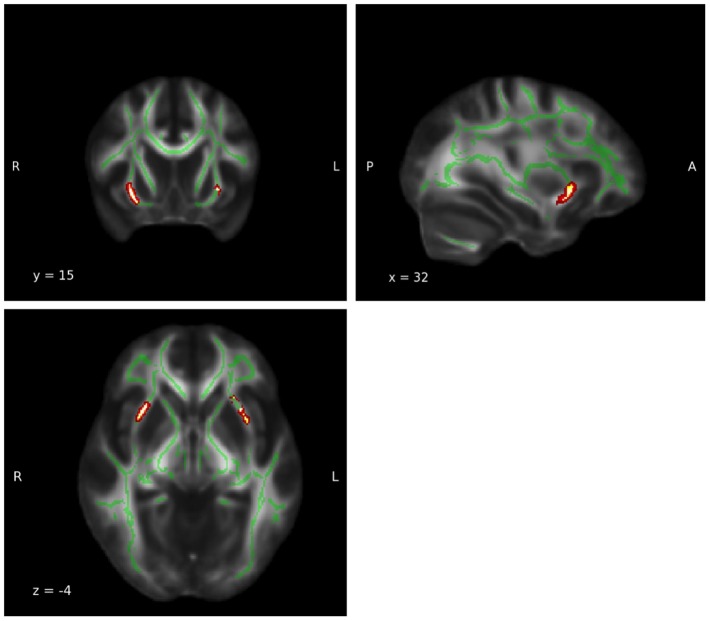
**Goup difference: Insula**. A significant FA group difference in which the meditators displayed higher FA values was detected in the white matter adjacent to the left (MNI coordinates: x = −25, y = 19, z = −5; PFWE-corrected = 0.005) and right (MNI coordinates: x = 30, y = 14, z = −4; PFWE-corrected = 0.006) insula. The red and yellow areas represent the group difference results for the insula. The green areas represent the WM skeleton of all participants.

Thalamus and insula, which showed the most robust results illustrate the outcome of the group difference analysis in Figures 4, 5.

### Main effect age

The results for the main effect age revealed significant clusters only in the right thalamus, in the left insula (*p* < 0.05, FWE corrected) and a notable trend in the right insula (*p* = 0.052, power = 0.742, FWE corrected; power calculated using G-Power Faul et al., 2009). Uncorrected results on the other hand show a negative age effect in all five ROIs (Tables in Supplementary Material).

### FA whole brain analysis

**Figure 6:**
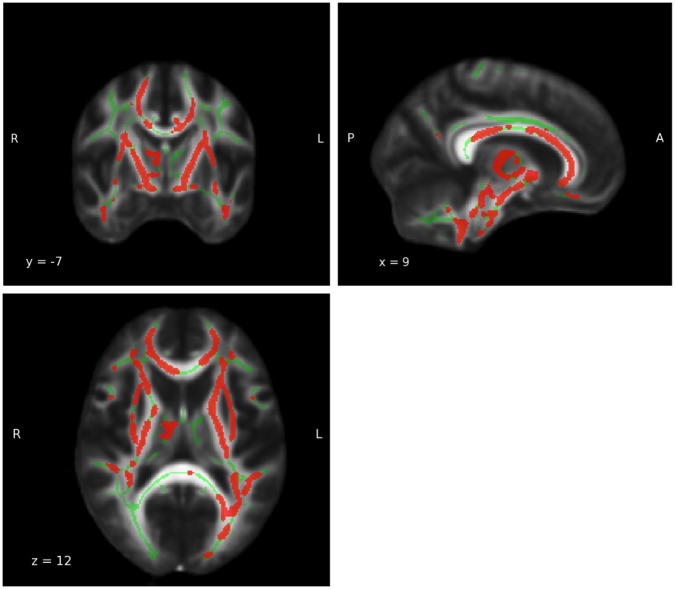
**Whole brain main effect age**. For results of the whole brain analysis a significant and extensive main effect age was observed in a large part of the white matter (WM) (MNI coordinates: x = 9, y = −7, z = 12; P_FWE−corrected_ = 0.05). The red area represents the results for main effect age. The green areas represent the WM skeleton of all participants.

A subsequent whole brain analysis revealed a clear and substantial decline of FA measures with age for the main effect age (*p* < 0.05, FWE corrected; see Figure 6). No other statistically significant results were found for group-by-age interaction and group difference.

## Discussion

In order to investigate FA group differences and the group-by-age interaction between experienced meditators and non-meditators, we employed diffusion tensor imaging on WM adjacent to five key brain regions.

We expected to find higher FA values in the WM connected to the thalamus, insula, amygdala, hippocampus, and ACC and a lower age-related FA decline for the meditators' group.

For the group contrast we found FWE-corrected, significantly higher FA values for four of the five a priori selected ROIs (thalamus, insula, amygdala, hippocampus) in the meditators compared with controls. The ACC also showed the same trend but the results are reported without correction for multiple comparisons. For the group-by-age interaction meditators did not display the typical natural FA decline which was found in non-meditators across all ROIs. This may suggest a tendency for the practitioners of MM to maintain a higher level of FA values in these areas. However, an opposite trend was found in the right thalamus, right amygdala, and right hippocampus.

Although a direct relationship between FA and WM structure is difficult to prove, FA alterations are believed to reflect a change in the diffusion of water molecules along fiber bundles. Generally, water molecules display a better diffusivity coefficient in the presence of a greater axon density, enhanced axonal membrane integrity, larger axonal diameter, greater myelination and a more coherent orientation (Thomason and Thompson, 2011). A higher FA value in a specific WM tract is, therefore, commonly interpreted as an indication of increased connectivity.

In contrast, reduced FA has been found in the regular aging process (Bennett et al., 2010; Burzynska et al., 2010; Michielse et al., 2010; Westlye et al., 2010; Lebel et al., 2012; Sala et al., 2012) and in several neurological or psychiatric disorders (Mueller et al., 2012a,b).

Our results suggest that the regular and prolonged practice of MM may have an impact on the structure of WM fibers adjacent to the thalamus due to the increased activation of this region during meditation. Among other functions the thalamus is considered to play a part in relaying sensory information to the cerebral cortex. This appears to be consistent with the fact that the practice of MM places a particular focus on moment to moment sensory perception although whether the MM practitioner actually increases sensory sensitivity during meditation needs to be objectively measured. Our findings therefore support previous studies such as (Luders et al., 2009) that reported an increased GM in this region and (Newberg et al., 2001, 2010) in which a significant increase in regional cerebral blood flow was observed in the thalamus.

The insula has been found to be highly involved in body awareness, which, in turn, is considered to be a relevant aspect of MM. Hölzel et al. (2008), for instance, have found a greater GM concentration in the right anterior insula, which the authors consider to have an influence on interoceptive awareness. The involvement of this region in meditation is also supported by Lazar et al. (2005) in which the right anterior insula was found to be thicker in meditation participants. A recent study has provided some evidence in which the insular cortex also appears to play a role in emotional awareness (Gu et al., 2013). The authors propose a model in which the insular cortex is essential for integrating bottom-up interoceptive signals with top-down predictions in order to generate an awareness state and provides a point of reference for autonomic reflexes. The DTI results of our group difference analysis seem to extend previous functional MRI and VBM findings to the microstructure of the WM around the insula, suggesting that this region may also be affected by the practice of MM.

It has been shown that the amygdala and the ACC are involved in emotion regulation. Hölzel et al. (2007) suggested that mindfulness meditators facilitate the regulation of their emotions by increasing the activation of the rostral ACC and the dorso-medial prefrontal cortex. In addition, Hölzel et al. (2010) observed a decrease in right basolateral amygdala GM density in stressed individuals in a longitudinal study. Various other studies have reported that the deactivation of the amygdala is associated with emotion regulation (Beauregard et al., 2001; Schaefer et al., 2002). Our results demonstrate significantly higher FA values in the amygdala, and a statistical trend for the ACC. If we assume that the downregulating effect of the ACC on the activation of the amygdala is increased by the practice of MM (Hölzel et al., 2013), the results may suggest an impact on the WM fibers connecting the two regions for which axonal membrane integrity, axonal diameter, level of myelination, and orientation might be positively affected.

The ACC and amygdala, in combination with the insula are also considered to play a role in the regulation of pain (Grant et al., 2011; Duerden and Albanese, 2013). Studies like (Morone et al., 2008; Grant and Rainville, 2009) have likewise produced some evidence that the practice of meditation could influence the perception and the acceptance of pain. Moreover, Grant et al. (2010), Gard et al. (2012), and Zeidan et al. (2012) suggested that meditators are able to apply unique brain mechanisms which help pain regulation. Grant et al. (2011) observed that ACC, thalamus, and insula, showed a stronger activation in meditators than in controls and activity during pain was reduced in the prefrontal cortex, amygdala, and hippocampus areas. The results of our study therefore appear to support the hypothesis that the training of the mind with MM has an effect on pain regulating mechanisms. This in turn may result in an alteration of the WM and the connectivity in the involved regions.

In the group-by-age interaction, the areas of the right hippocampus, right amygdala, and right thalamus exhibited distinct clusters in which meditators and controls displayed both weaker and stronger slopes, suggesting that different parts of these regions are influenced in different ways. One plausible explanation for these results could be the functionally distinct structures of these ROIs. Previous findings support this view with regard to the hippocampus and have demonstrated that its ventral and dorsal parts have different functions (Fanselow and Dong, 2010). Fanselow and Dong (2010) suggest that the dorsal hippocampus performs primarily cognitive functions, while the ventral part relates to stress, emotion, and affect. Our results seem to support this theory showing that meditators display a stronger slope in the dorsal hippocampus (cognitive functions) and non-meditators display a stronger slope in the ventral part (stress, emotion, affect functions; see Figure 3).

Similarly, the literature describes the amygdala as a brain region composed of several nuclei which relate to different functions (Sah et al., 2003; Pabba, 2013). In our results, the cluster in the right amygdala of the control group, which displays a weaker negative slope for FA measures in the group-by-age interaction, could point to the differentiation in functionality of the various nuclei which meditation may only be able to influence selectively.

The thalamus also incorporates several nuclei with distinct functions which range over motor control, sensory relaying to the cerebral cortex, sleep and awake state control and consciousness (Morel et al., 1997; Van der Werf et al., 2002; Jang et al., 2014). As with the amygdala and hippocampus, the practice of MM may affect different nuclei of the thalamus in distinct ways.

## Limitations

Although all meditators reported to practice MM, meditation styles, and meditation routines varied greatly. Many experienced meditators combine meditation techniques in their regular practices and throughout their lifetime. It was therefore not possible in this study to test for structural heterogeneities of a specific meditation technique.

Meditation experience is an additional important factor which is undoubtedly very difficult to control. Although participants stated their experience in terms of years of practice, this indicator is not very accurate in determining the real extent of each participant's meditation training. Daily length and intensity of practice tend to be subjective measures and vary over time. These factors may have, therefore, affected the outcome of the investigation. Moreover, as the design of this study was cross-sectional in nature, it was not possible to determine the causal relationship between the practice of MM and brain WM microstructure. The possibility that the brain of meditators was different before meditation cannot be excluded. Longitudinal studies will therefore be necessary to determine causation.

In order to confirm the results of this study and demonstrate their behavioral and clinical relevance, additional WM investigations are required. A tractography analysis could provide supplementary information about connectivity between these ROI. Future research should also include neurocognitive measures in order to determine whether preservation of WM microstructure in meditators correlates with the preservation of mental abilities, altered behaviors, and subjective well-being.

## Conclusions

This study concludes, as was hypothesized, that apart from the right amygdala, right hippocampus, and right thalamus which showed an opposite trend in the group-by-age interaction (probably due to their nuclei subdivision), meditators showed a weaker negative slope for FA values compared to non-meditators. This could be an indication that the regular practice of MM may contribute to the preservation of fiber integrity in the WM around the selected ROIs. This stands in contrast to a natural steady age-related decline which occurred in the control group. The group difference results present a consistent picture in one-direction (MED>CON) and thus express a clear indication of a probable MM effect on the microstructure of the WM. These results are in line with our expectations and mostly support previous findings. The current data suggest that the greater FA values displayed by meditators in the group comparison could be associated with the practice of MM and might be a sign of WM fiber integrity. Further research is needed to elucidate possible neuroplastic changes and training effects that could be responsible for these findings.

### Conflict of interest statement

The authors declare that the research was conducted in the absence of any commercial or financial relationships that could be construed as a potential conflict of interest.
