# Supplementary material for: Effects of Long-Term Mindfulness Meditation on Brain's White Matter Microstructure and its Aging
Source: Front Aging Neurosci. 2016 Jan 14;7:254. doi: 10.3389/fnagi.2015.00254 (PMC4712309; doi:10.3389/fnagi.2015.00254)
Supplement: Supplementary file 1 [file Table1.doc]

***
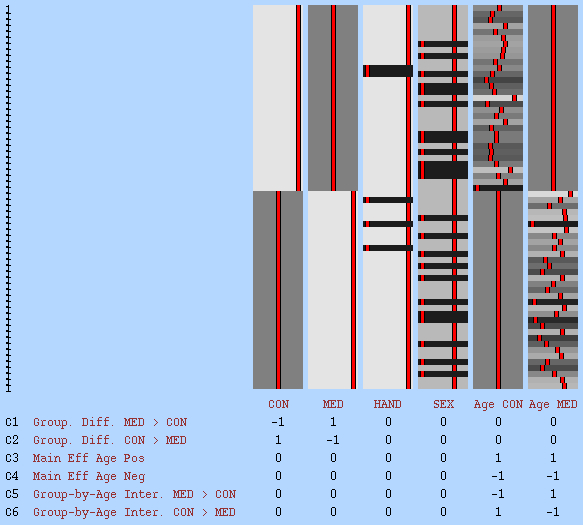
***

## ***Generalized linear model (GLM)***

*Summary of the GLM design utilized for the analysis of the DTI data. The design included investigations for main effects, group difference and age and group-by-age interaction.*
