# Supplementary material for: Effects of Long-Term Mindfulness Meditation on Brain's White Matter Microstructure and its Aging
Source: Front Aging Neurosci. 2016 Jan 14;7:254. doi: 10.3389/fnagi.2015.00254 (PMC4712309; doi:10.3389/fnagi.2015.00254)
Supplement: Supplementary file 2 [file Table2.doc]

***Age interaction 2: CON > MED***

*Group-by-age interaction in which controls exhibit a weaker slope in the relationship between FA and age compared to meditators (CON > MED). Only the Right thalamus, right amygdala and right hippocampus showed a significant interaction (p<0.05, uncorrected).*

**Anatomical Coordinates Cluster**

**region x y z No. voxels P-Value**

**Thalamus**

Left - - - - -

Right 18 -32 9 23 0.023

**Insula**

Left - - - - -

Right - - - - -

**Amygdala**

Left - - - - -

Right 24 -11 -10 20 0.015

**Hippocampus**

Left - - - - -

Right 27 -31 5 118 0.005

19 -31 6 17 0.017

**ACC**

Left - - - - -

Right - - - - -
