# Supplementary material for: Effects of Long-Term Mindfulness Meditation on Brain's White Matter Microstructure and its Aging
Source: Front Aging Neurosci. 2016 Jan 14;7:254. doi: 10.3389/fnagi.2015.00254 (PMC4712309; doi:10.3389/fnagi.2015.00254)
Supplement: Supplementary file 3 [file Table3.doc]

***Main Effect AGE Negative***

*Main effect age (decreasing FA) for both meditators and controls. Right thalamus and left insula showed statistically significant results (p<0.05, FWE corrected) and the right insula a clear trend (p<0.1, FWE corrected). Uncorrected trends in all other regions are also reported (p<0.05, uncorrected).*

**Anatomical Coordinates Cluster**

**region x y z No. voxels P-Value**

**Thalamus**
Left 0 -2 9 53 0.001^uc^

-24 -32 10 30 0.003^uc^

-10 -5 9 16 0.004^uc^

-4 -15 -5 13 0.003^uc^

-3 -6 10 9 0.001^uc^

-16 -15 15 8 0.017^uc^

Right 4 -4 9 28 0.014**^c^**

12 -9 13 20 0.029**^c^**

**Insula**

Left -30 -2 16 24 0.034**^c^**

Right 30 8 13 25 0.052**^ct^**

**Amygdala**

Left -23 -15 -5 72 0.002^uc^

-12 -11 -12 16 0.02^uc^

Right 13 -6 -9 98 0.001^uc^

34 0 -25 91 0.002^uc^

25 -18 -7 18 0.013^uc^

**Hippocampus**

Left -22 -31 -3 118 0.005^uc^

-24 -11 -10 19 0.005^uc^

-26 -17 -29 17 0.012^uc^

-17 -45 -9 14 0.014^uc^

Right 23 -30 -2 70 0.008^uc^

18 -15 -9 22 0.003^uc^

**ACC**

Left -20 42 13 27 0.004^uc^

Right 21 38 22 27 0.005^uc^

21 40 11 24 0.004^uc^

10 36 -16 20 0.002^uc^

12 23 19 18 0.011^uc^

19 45 -2 18 0.008^uc^

*c=corrected (p<0.05)*

*ct=corrected trend (p<0.1)*

*uc=uncorrected*
